# Intratumoral microbiota: roles in cancer initiation, development and therapeutic efficacy

**DOI:** 10.1038/s41392-022-01304-4

**Published:** 2023-01-16

**Authors:** Li Yang, Aitian Li, Ying Wang, Yi Zhang

**Affiliations:** 1 Biotherapy Center, The First Affiliated Hospital of Zhengzhou University, Zhengzhou, 450052 China; 2 School of Life Sciences, Zhengzhou University, Zhengzhou, 450001 P.R. China; 3 State Key Laboratory of Esophageal Cancer Prevention & Treatment, Zhengzhou, 450052 China

**Keywords:** Tumour immunology, Microbiology, Tumour immunology

## Abstract

Microorganisms, including bacteria, viruses, fungi, and other eukaryotes, play critical roles in human health. An altered microbiome can be associated with complex diseases. Intratumoral microbial components are found in multiple tumor tissues and are closely correlated with cancer initiation and development and therapy efficacy. The intratumoral microbiota may contribute to promotion of the initiation and progression of cancers by DNA mutations, activating carcinogenic pathways, promoting chronic inflammation, complement system, and initiating metastasis. Moreover, the intratumoral microbiota may not only enhance antitumor immunity via mechanisms including STING signaling activation, T and NK cell activation, TLS production, and intratumoral microbiota-derived antigen presenting, but also decrease antitumor immune responses and promote cancer progression through pathways including upregulation of ROS, promoting an anti-inflammatory environment, T cell inactivation, and immunosuppression. The effect of intratumoral microbiota on antitumor immunity is dependent on microbiota composition, crosstalk between microbiota and the cancer, and status of cancers. The intratumoral microbiota may regulate cancer cell physiology and the immune response by different signaling pathways, including ROS, β-catenin, TLR, ERK, NF-κB, and STING, among others. These viewpoints may help identify the microbiota as diagnosis or prognosis evaluation of cancers, and as new therapeutic strategy and potential therapeutic targets for cancer therapy.

## Introduction

Humans contain a large number of microorganisms, which play critical roles in human health.^1^ The human commensal microbiome includes bacteria, viruses, fungi, and other eukaryotic species,^2^ which can inhabit many sites in the human body, including the mouth, gastrointestinal tract, reproductive system organs, and skin.^3,4^

Many studies of the human microbiome indicate that the microbiota differs between healthy and diseased individuals. In particular, the microbiota has a close relationship with cancer; it affects carcinogenesis in the human body.^5^ Oncoviruses induce tumorigenesis by integrating oncogenes into the human host genome. Interestingly, different intratumoral microbial components, which are significantly correlated with cancer initiation and development, have been found and evaluated in several kinds of tumor tissues. Garrett et al.^6^ reported three ways in which the microbiota may lead to tumor progression and development: (1) changing the balance of cell proliferation and apoptosis, (2) reprogramming the immune system and responses, and (3) affecting the metabolism of host-secreted factors, foods, and drugs.

Many studies have shown that the gut microbiota is essential for the regulation of host immune responses. However, the intratumoral microbiota may also play a key role in shaping the local immune responses of the tumor microenvironment, which further affects tumor progression. The intratumoral microbiota play different roles in antitumor immunity: by either enhancing or decreasing antitumor immune responses and inducing different immunotherapy efficacies and outcomes.^7,8^

In this review, we describe the intratumoral microbiota in a comprehensive way, including the history and milestones, the origin, the diversity of intratumoral microbiota, the relationship between intratumoral and gut microbiota, the effect of the intratumoral microbiota on cancer development, antitumor immunity and therapeutic efficacy, and the usage of intratumoral microbiota for therapy, diagnosis and prognosis of cancers. These findings may help identify new therapeutic strategies and targets of intratumoral microbiota for cancer therapy.

## History and milestones of intratumoral microbiota

**Fig. 1:**
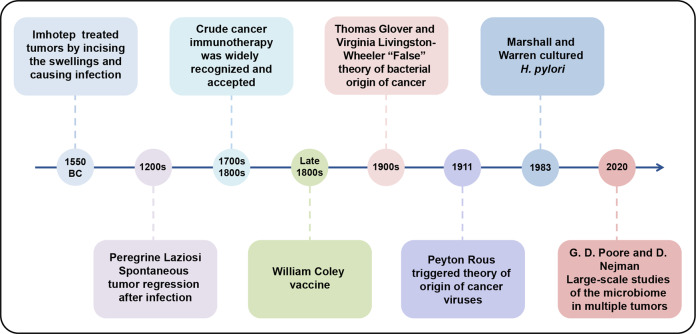
Timeline of the history and milestones of intratumoral microbiota. The eight key research milestones of intratumoral microbiota were retrospectively summarized from 1550 BC to present day

The key research milestones of intratumoral microbiota were retrospectively summarized (Fig. 1). The history of microbes in tumors can be traced back to as early as 1550 BC, when the Egyptian physician Imhotep (2600 BC) treated tumors by incising swellings and then causing infection.^9,10^ In the 13th century, Peregrine Laziosi (1265–1345) had a huge growth on his tibia and developed a severe infection after amputation, but the cancer never returned, and centuries later he was named the patron saint of cancer patients.^11^ Subsequent reports of spontaneous tumor regression following infection followed, and by the 18th and 19th centuries, this crude cancer immunotherapy was widely recognized and accepted.^12^ It was not until the late 1800s that William Coley successfully treated sarcoma patients with a vaccine made of two inactivated bacteria (*Streptococcus pyogenes* and *Serratia marcescens*) by direct injection into the tumor site, which was promoted as the first intentional demonstration of immunotherapy and promoted.^10,13,14^ In the 1900s, Thomas Glover and Virginia Livingston-Wheeler claimed that bacteria could be grown from tumors and suggested a common bacterial origin for cancer, but ultimately proved their theories incorrect.^14–16^ In 1911, Peyton Rous discovered that the breast tumor filtrate of chickens can lead to a transmissible sarcoma, which may be caused by a minute parasitic organism, triggering the theory of the origin of cancer viruses.^17^ In the following decades, people have gradually discovered viruses that can induce carcinogenesis, such as the Epstein-Barr virus (EBV), Kaposi’s sarcoma-associated herpesvirus, human papilloma virus, human T-cell lymphotropic virus, hepatitis B virus (HBV), hepatitis C virus (HCV), and Merkel cell polyomavirus (MCPyV). In 1983, Marshall and Warren cultured *Helicobacter pylori* and demonstrated its role in peptic ulcers,^18,19^ and subsequent studies proved that this bacterium can cause stomach cancer, which sparked a wave of research on how bacteria can cause cancer. Since the 21st century, with the development of sequencing technology, more and more articles have reported the existence of microbiota in tumors and revealed their importance in the tumor microenvironment and regulation of treatment outcomes.^3,20–28^ The widespread use of the Next Generation Sequencing has further advanced the study of intratumoral microbiota. In 2020, two large-scale studies on the microbiota in multiple tumors were reported. Poore et al. analyzed the diverse intratumoral microbiota in more than 30 cancers and proposed a new diagnostic tool based on microbiota for cancer.^29^ Shortly thereafter, Ravid Straussman’s team conducted the first comprehensive analysis of seven tumor microbiomes, which providing the intratumoral spatial distribution of these microbiota and imaging evidence of intracellular localization.^8^ In 2022, the team again revealed the distribution of fungi in 35 cancers, their localization in cells and synergistic effects with bacteria.^30^ Coincidentally, at the same time, Dohlman et al. analyzed The Cancer Genome Atlas data to discover disease-related fungi in cancers of the gastrointestinal tract, lungs, breast, head and neck, and studied the role of fungal DNA in diagnosis and prognosis.^31^

## The characteristics of intratumoral microbiota

In recent years, the analysis of intratumoral microorganisms has identified microorganisms that are found in the complex system of 3.8 × 10^13^ bacteria that colonize the intestine, suggesting that intestinal microbes can enter the tumor site through circulation to colonize the tumor, but not all intratumoral microorganisms are derived from the intestine. Therefore, we will discuss the characteristics of intratumoral microbiota including their origin and diversity, and the relationship between intratumoral and gut microbiota.

### Origin of intratumoral microbiota

Recent evidence has shown that the potential sources of intratumoral microorganisms can be classified into three categories:^32^ (1) Through mucosal barrier sources, including in colorectal cancer, pancreatic cancer and other digestive tract tumors, lung cancer, cervical cancer, etc.; these organs have a cavity that is externally exposed, and microorganisms colonizing the mucosa may invade the tumor due to mucosal destruction during tumorigenesis; (2) From adjacent normal tissues, this is based on a study that found that some bacteria are also present in organs that were originally thought to be sterile, and the bacterial composition in tumor tissues is highly similar to adjacent normal tissues. Furthermore, the immunosuppression and hypoxic microenvironment of tumors enhances microbial colonization. However, the source of microorganisms in normal tissues is not clear, and it may also spread from the tumor site, so this idea needs more research evidence for confirmation; (3) Through hematogenous spread, where microorganisms from the mouth, intestines, and other potential sites may be transported through the blood to the tumor site and infiltrate the tumor through damaged blood vessels. A study of canine breast tumors showed the presence of *Bacteroides* in the tumor microbiome, as well as in the mouth and gut, suggesting that the microorganism may spread from the mouth to the intestine, and eventually to distant tumor tissue.^33^ When colorectal cancer occurs, *Escherichia coli* damages the gut vascular barrier, enters the blood circulation and subsequently colonizes the liver, inducing the formation of a pre-metastatic microenvironment, and promoting liver metastasis.^34^ It has been observed that all tumor-related bacteria and fungi are mostly located in cells, including cancer cells and immune cells, which increases the possibility that microorganisms do not enter the tumor or adjacent tissues in a free state, but are transported there in the form of fragments or intact cells through cell migration,^30,35^ but it cannot be ruled out that bacteria in the blood vessels directly infect the tumor site.

### Diversity of intratumoral microbiota

**Fig. 2:**
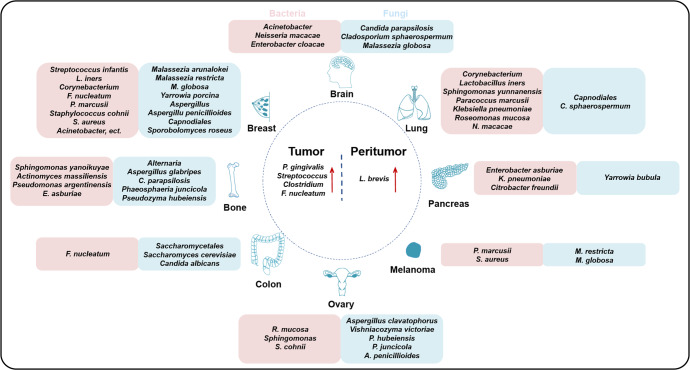
The diversity of intratumoral microbiota. Several tumors have been closely correlated with microbial infections. Each tumor type, including lung, breast, pancreas, ovary, brain, bone tumors, and melanoma, has a distinct bacterial and fungal composition. Moreover, a distinct community of microbiota between the tumor and peritumor tissues has been found

As there may be multiple sources of microbes within tumors, it can be speculated that the microorganisms compositions of different cancer types are diverse, as confirmed by the large-scale study of the microbiome by Ravid Straussman’s team (Fig. 2, Table 1). Their study on the tumoral microbiome of seven tumor types, namely lung, breast, pancreas, ovary, brain, bone tumors, and melanoma, revealed that each tumor has a different microbiome composition.^8^

**Table 1:** The major features of intratumoral microbiota

Tumor type	Bacterial compositions	Effect 1		Ref. 1	Fungal compositions	Effect 2		Ref. 2
Intestinal cancer	*E. coli*	Initiation of cancers	DNA mutations	^68^	*Saccharomycetales, S. cerevisiae, C. albicans*	No results posted		^30^
	*B. fragilis*			^74,76^				
	*C. jejuni*			^75^				
	*H. pylori*		Activating carcinogenic pathways	^79^				
	*S. typhi*			^80,81^				
	*F. nucleatum*	Promoting tumor progression	Promoting chronic inflammation	^62^				
	*P. gingivalis*			^86^				
	*Bifidobacterium*	Enhancing antitumor immune response	STING signaling activation	^93^				
	*H. hepaticus*		TLS production	^101^				
	*Fusobacterium*	Decreasing antitumor immune response	Upregulation of ROS	^90,102^				
	*Methylobacterium*		T cell inactivation	^107^				
Glioblastoma	*Acinetobacter, N. macacae, E. cloacae*	No results posted		^8^	*C. parapsilosis, C. Sphaerospermum, M. globose*	No results posted		^30^
Pancreatic ductal adenocarcinoma	*E. asburiae, K. pneumonia, C. freundii*	No results posted		^8^	*Y. bubula*	No results posted		^30^
	*P. gingivalis*	Promoting tumor progression	Promoting chronic inflammation	^66^	*Malassezia*	Promoting tumor progression	Activation complement system	^27^
	*Saccharopolyspora, Pseudoxanthomonas, Streptomyces*	Enhancing antitumor immune response	T cell activation	^43^				
Melanoma	*P. marcusii, S. aureus*	No results posted		^8^	*M. restricta, M. globose*	No results posted		^30^
	*A. muciniphila*	Enhancing antitumor immune response	STING signaling activation	^44^				
	*Lachnoclostridium*		T cell activation	^95^				
	*Bifidobacterium*		NK cell activation	^100^				
Lung cancer	*Corynebacterium, L. iners, S. yunnanensis, P. marcusii, K. pneumonia, R. mucosa, N. macacae*	No results posted		^8^	*Capnodiales, C. sphaerospermum*	No results posted		^30^
Breast cancer	*S. infantis, L. iners, Corynebacterium, F. nucleatum, P. marcusii, S. cohnii, S. aureus, Acinetobacter*	No results posted		^8^	*M. arunalokei, M. restricta, M. globosa, Y. porcina, Aspergillus, A. penicillioides, Capnodiales, S. roseus*	No results posted		^30^
	*Staphylococcus, Lactobacillus, Streptococcus*	Promoting tumor progression	Initiating metastasis	^87^				
	*Clostridiales*	Enhancing antitumor immune response	T cell activation	^94^				
Ovarian cancer	*R. mucosa, Sphingomonas, S. cohnii*	No results posted		^8^	*A. clavatophorus, V.victoria, P. hubeiensis, P. juncicola, A. penicillioides*	No results posted		^30^
Bone cancer	*S. yanoikuyae, A. massiliensis, P. argentinensis, E. asburiae*	No results posted		^8^	*Alternaria, A. glabripes, C. parapsilosis, P. juncicola, P. hubeiensis*	No results posted		^30^

Recently, Ravid Straussman’s team uncovered the fungal microbiome atlas in 35 cancer types and found that there were significant differences in the richness of the microbiome in different cancer types. The results showed that fungi appeared in all studied cancer types, while specific fungal species and their localized cell types were related to the cancer types. However, bacteria predominated tumor microbial communities, while fungi were less abundant, and a similar community combination was found in adjacent normal tissues.^30^ Some microorganisms are present in a variety of tumors, but their proportions vary among different cancer types. Recent findings by Galeano Nino et al. further uncovered the spatial and population heterogeneity of intratumoral microbiome.^36^ Several reviews have compiled detailed tables to clarify the microbial composition of different cancer types.^32,37^ Due to recent breakthroughs in research of intratumoral microbiota compositions, we have summarized the main bacterial and fungal compositions of eight different cancers published in large-scale cohort studies in recent years (Fig. 2, Table 1).^8,30^

Besides, the microbial distribution also differs between tumor and peritumoral tissues. A study on breast cancer showed that normal and tumor tissues had distinct microbial communities.^38^ The abundance of *Porphyromonas gingivalis* and oral microbiota, such as *Clostridium* and *Streptococcus*, in esophageal carcinoma and gastric cancer tissues, respectively, were significantly higher than those in paired peritumor tissues.^39,40^ Meanwhile, *Lactobacillus brevis* was less enriched in tumor tissues compared to adjacent normal tissues.^40^ Other studies have reported that *Fusobacterium nucleatum* was enriched in colorectal cancer tissues, but not in adjacent normal tissues (Fig. 2).^22,41^

### Connection between intratumoral and gut microbiota

There is some correlation between intratumoral and gut microbiota. Firstly, as previously mentioned, intratumoral microbiota can arise from gut microbiota as microorganisms from the intestines could be transported through the blood to tumor sites.^32^

Secondly, several studies have confirmed that both intratumoral microbes and gut microbes have a regulatory effect on the tumor microenvironment.^42–46^ It is now believed that the gut microbiota can modulate the hematopoietic and non-hematopoietic components of the intestinal epithelial barrier, activate primary and secondary lymphoid organs, and ultimately regulate the tumor microenvironment. This immune-mediated interaction and collective feedback loop is defined as the immuno-oncology microbiome axis.^14^ These reports have shown that gut microbes indirectly affect the tumor microenvironment through their metabolites or the immune system, which may further affect the composition and function of the intratumoral microbiota. However, the effects of the interaction between the two on the tumor microenvironment is still unclear.

Furthermore, the intratumoral microbiota may regulate host immune responses, similarly to the gut microbiota. The antitumor efficacy of the immune checkpoint blockade (ICB) and antitumor immune responses depend on distinct gut microbiota.^47–50^ Recent literature reports suggest a link between the abundance of intratumoral microorganisms. A large-scale cohort study of bacterial groups, fungal groups and immune groups in various tumors showed a significant positive correlation between fungal and bacterial abundance in bone, breast, glioblastoma multiforme and lung tumors, suggesting that there was no competitive relationship between fungi and bacteria. In-depth analysis revealed three fungal-bacteria-immune cell symbiotic relationships driven by fungi, called “mycotypes”, and the types of relationships related to different host immune responses.^30^ This suggests that different intratumoral-based microbiome interactions may trigger different host immune responses, and indicates that gut microbes may also interact with intratumoral microbes. But does this effect require gut microbes to migrate into the tumor directly? Do gut microbes communicate with intratumoral microorganisms through their metabolites or the immune system? Or can intratumoral microorganisms affect the composition and metabolism of intestinal microorganisms? More research is needed to explore the interaction between the intratumoral and gut microbiomes.

## Effects of the intratumoral microbiota on cancer development

Increasing evidence has shown that several tumors are closely correlated with infection by microorganisms, including bacteria, viruses, and fungi.^20,26,51–55^ Intratumoral microbiota are also closely associated with cancer development, including the positive and negative effects on tumor progression.^24,56^

### Initiation and progression of cancers

**Fig. 3:**
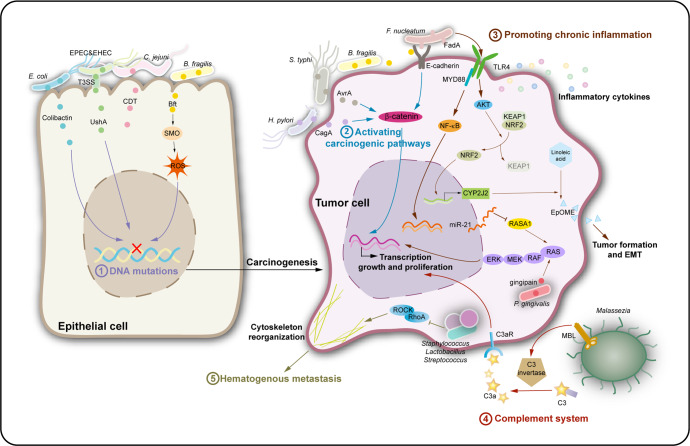
The mechanisms involved in the intratumoral microbiota-promoted tumorigenesis and cancer development. The intratumoral microbiota may contribute to promotion of the initiation and progression of cancers by DNA mutations, activating carcinogenic pathways, promoting chronic inflammation, complement system, and initiating metastasis. (1) DNA mutations: Toxins produced by intratumoral microbiota can directly damage host cell DNA, or indirectly damage through ROS production, which leads to genetic mutations and carcinogenesis. (2) Activating carcinogenic pathways: Some intratumoral microbiota can produce effectors (CagA and AvrA) to activate the β-catenin signaling pathway in the host cell, which induces cell growth and proliferation; Bft derived from *B. fragilis* stimulates E-cadherin cleavage, and FadA on the surface of *F. nucleatum* binds to E-cadherin on colon cancer cells, thereby activating the β-catenin signaling pathway. (3) Promoting chronic inflammation: Intratumoral microbiota can bind to pattern recognition receptors to produce a variety of cytokines and activate the NF-κB signaling pathway, thereby forming a positive cycle, leading to chronic inflammation and promoting tumor progression. At the same time, *F. nucleatum* can activate the TLR4/MYD88/NF-κB signaling pathway to increase miR-21 and inhibit RASA1 expression in colorectal cancer cells, thereby triggering the RAS signaling pathway to result in an increase of transcription genes related to growth and proliferation. In addition, *F. nucleatum* can activate TLR4 signaling pathway to increase CYP2J2, and then catalyze linoleic acid to promote the production of 12,13-EpOME, which leads to EMT and tumor formation. Moreover, *P. gingivalis* activates the MAPK signaling pathway through gingipain to promote cancer cell proliferation. (4) Complement system: In pancreatic duct adenocarcinoma, *Malassezia*’s fungal wall glycans can be recognized by MBL in the tumor environment, which activates C3 invertase to promote cell proliferation, motility, and invasiveness. (5) Initiating metastasis: *Staphylococcus*, *Lactobacillus*, and *Streptococcus* in breast cancer cells can inhibit the RhoA-ROCK signaling pathway to reshape the cytoskeleton and help tumor cells resist mechanical stress in blood vessels and promote hematogenous metastasis

Intratumoral organisms are involved in tumorigenesis and cancer development.^57–66^ Microbiota also have a distinct effect on carcinogenesis in each organ, as both the characteristics of host and microbial genotypes affect the susceptivity to and promotion of cancer.^5^ The microorganisms promote tumorigenesis and development through a variety of mechanisms (Fig. 3, Table 1).

#### DNA mutations

Inducing DNA mutations is one of the carcinogenic mechanisms of microorganisms (Fig. 3). Oncoviruses are key driving factors in the initiation of more than 10% of human cancers.^67^ It is commonly known that oncoviruses lead to cancer, such as HBV and human papilloma virus, by integrating the viral genome into the host chromosome, causing host cells to transform and divide out of control, resulting in cell malignancy. Some carcinogenic bacteria can also damage host DNA in a wide variety of ways, causing genetic mutations that lead to tumorigenesis (especially in gastrointestinal tumors). Since 2006, scientists have found that the pks locus-encoded colibactin expressed by *E. coli* can cause DNA double-strand breaks, leading to DNA damage and carcinogenesis.^68^ Subsequently, a number of studies have supported and confirmed the theory.^69,70^ In 2020, one study directly demonstrated that Pks^*+*^ *E. coli* can cause genetic mutations in colorectal cancer cells.^71^ Recent studies have shown that adherent pathogenic bacteria (such as *Enteropathogenic E. coli* and *Enterohemorrhagic E. coli*), which can cause transient diarrhea, can interact with intestinal epithelial cells through their type 3 secretion system, and inject genotoxin-UshA that destroys the DNA of intestinal epithelial cells, causing carcinogenesis.^72^ Intestinal mucosal analysis of patients with familial adenomatosis showed the enrichment of *E. coli* and *Bacteroides fragilis*, which together colonize epithelial cells and produce interlukin-17 (IL-17) resulting in massive DNA damage to the epithelium.^73^ The toxin (Bft) secreted by *B. fragilis* and cytolethal distending toxin (CDT) produced by *Campylobacter jejuni* can also damage DNA,^74,75^ possibly by upregulating spermine oxidase (SMO) in intestinal epithelial cells to induce reactive oxygen species (ROS) production.^76^ Inhibition of DNA oxidative damage reduces microbial-induced colitis-associated colorectal cancer.^77^

#### Activating carcinogenic pathways

Microbes can also promote tumor development by activating carcinogenic pathways (Fig. 3). The β-catenin signaling pathway is an essential cancer intrinsic signal.^78^ Multiple pathways for microbial activation of β-catenin signaling have been reviewed in the literature.^6^ For instance, CagA produced by *H. pylori* is directly injected into the cytoplasm of the host cell and activates β-catenin signaling, driving gastric cancer;^79^ AvrA secreted by *Salmonella typhi* also activates β-catenin signaling;^80,81^ The adhesion molecule FadA expressed on the surface of *F. nucleatum* binds to E-cadherin on host cells,^82^ and the Bft produced by *B. fragilis* can also stimulate E-cadherin cleavage, thereby activating β-catenin.^83^

#### Promoting chronic inflammation

Persistent chronic inflammation can form a tumor-permissive milieu in multiple tissues, thus, is one of the culprits that lead to tumors.^84^ Microbiota can induce chronic inflammatory responses resulting in tumorigenesis (Fig. 3). Numerous studies have shown that microorganisms in tumors can bind to pattern recognition receptors, producing a variety of cytokines, activating nuclear factor-κ-gene binding (NF-κB) signaling pathways, form a positive feedback cycle, inducing pro-inflammatory responses, and promoting tumor progression.^6^ *F. nucleatum* can induce colorectal cancer cell proliferation and migration, through activation of the toll-like receptor 4 (TLR4)/myeloid differentiation primary response gene 88 (MYD88)/NF-κB signaling pathway in tumor cells, increasing the expression of microRNA-21 (miR-21), thereby inhibiting RAS protein activator like 1 (RASA1), and activating the inherent RAS signaling of tumors, resulting in elevated transcription of genes related to growth and proliferation. At the same time, higher serum levels of inflammatory factors such as IL-17F, IL-21, IL-22, and MIP3A were also observed in mice infected with bacteria.^62^ It seems that TLRs play multiple roles in the interactions between microbes and tumors. Kong et al. found that *F. nucleatum* can activate TLR4 signaling, and increase CYP2J2 expression in cells, which then catalyze linoleic acid to produce more 12,13-EpOME, eventually leading to epithelial mesenchymal transformation (EMT) and promoting colorectal cancer formation and metastasis.^85^ In addition, studies have found that intracellular *P. gingivalis* can promote the proliferation of pancreatic and colorectal cancer cells, the latter mechanism may be facilitated by the activation of mitogen-activated protein kinase (MAPK) signaling by bacterial gingipains, as the bacteria that lack gingipains have a reduced ability to promote tumor growth.^66,86^

#### Complement system

Moreover, microbes can also contribute to tumor progression through the complement system (Fig. 3). Aykut et al. found that in pancreatic ductal adenocarcinoma, *Malassezia* is significantly enriched and its glycans of the wall can be recognized by mannose-binding lectin in the tumor environment to activate C3 invertase, resulting in an increase in C3a. Subsequently, C3a binds to C3aR on the surface of tumor cells to promote tumor proliferation, motility, and invasiveness.^27^ From the above results, it can be inferred that the impact of the microbiota on the activation of carcinogenic pathways occurs mostly in gastrointestinal-related cancers. Whether these pathways are also prevalent in other tumors and whether microbes could activate other cancer-associated signals requires more in-depth research to answer these questions.

#### Initiating metastasis

The latest evidence suggests that intratumoral microorganisms can initiate tumor metastasis (Fig. 3). Fu et al. reported that *Staphylococcus*, *Lactobacillus*, and *Streptococcus*, were enriched in breast cancer cells and can inhibit the RhoA-ROCK signaling pathway to reshape the cytoskeleton, thereby helping tumor cells resist mechanical stress in blood vessels to avoid damage, thus promoting tumor metastasis. Meanwhile, by using germ-free mice (without intestinal flora) and immunodeficient mice, it has been proven that tumor intracellular bacteria can play a role in promoting tumor metastasis independently of the intestinal flora and the immune system.^87^

From the above, it can be inferred that a variety of microorganisms present in different types of tumors can promote tumorigenesis, development, and metastasis of tumors through multiple signaling pathways. Based on existing research, we categorized the cancer-promoting pathways of microorganisms into the five types discussed above. These signaling pathways do not exist in isolation but are related to each other. Firstly, some microorganisms induce production of toxins or ROS species, which causes DNA mutation of host cells, thereby leading to tumorigenesis. At the same time, the tumor-intrinsic β-catenin signaling pathway is activated, which further promotes the malignant transformation of cells. This process may exacerbate cell deterioration because this signaling pathway is active in both host and tumor cells. In addition, some other tumor-intrinsic signaling pathways (e.g., MAPK) may be triggered by intratumoral microorganisms in a variety of ways, either directly, by stimulating upstream signaling, or by activating elements of the signaling pathway downstream from TLRs. In this process, the activation of NF-κB interacts with the production of related cytokines to form a positive feedback loop, leading to persistent chronic inflammation that is conducive to tumor growth. Activation of the complementary system also promotes tumor progression through complementary receptors on the surface of tumor cells. These changes in signaling pathway activation, induced by intratumoral microbiota, may affect tumor cell metabolism, leading to EMT and migration of tumor cells. During tumor hematogenous metastasis, the presence of intratumoral microorganisms regulates the cytoskeleton, thereby helping tumor cells to resist the blood fluid pressure and successfully achieve distant colonization. Therefore, the regulation of cancer biology by intratumoral microbiota is complex and diverse, and our existing knowledge of the signaling pathways through which these microorganisms exert their effects represents only the “tip of the iceberg”. Due to insufficient research, whether different microorganisms influence a specific subset of regulatory signaling pathways and whether this is tumor-type specific is unknown. In addition, it is possible that sequential effects exist, with multiple microorganisms influencing signaling at different stages of tumor development, jointly promoting tumor progression from various aspects.

### Preventing tumor progression

Although the intratumoral microbiota can promote tumor growth, it also plays a key role in inhibiting tumor progression (Table 1). A previous study identified the distinct microbial profiles within the tumor microenvironment of patients with pancreatic ductal adenocarcinoma with long-term survival, suggesting that the intratumoral bacterial signature could serve as a predictive biomarker for patients with good prognosis.^43^ There may be close crosstalk between intratumoral bacteria and the gut microbiota, leading to host immune responses and high intratumoral immune infiltration, which further slows tumor growth.^43^ In other words, “good” intratumoral microorganisms that attack tumor cells may be existed in patients with long survival, which could directly inhibit cell proliferation or cause tumor cell death. Unfortunately, there have been no similar reports so far. Some micobiota found in large-scale sequencing studies are reduced in tumor tissues compared to normal tissues. For example, the levels of *Cladosporium* are higher in normal and adjacent breast tissues than that in breast tumor tissues.^30^ Furthermore, fusarubin and anhydrofusarubin isolated from *Cladosporium* have been shown to obviously inhibit hematologic tumor cell proliferation and increase cell apoptosis at higher concentrations.^88^ Therefore, these data can provide a guide for searching “good” micobiota.

Regarding the interaction between intratumoral bacteria and cancer cells, there are still many key issues that need to be addressed, such as the relationship between intratumoral microorganisms and cancer cell proliferation, dormancy, stemness, metabolism, death, etc., which may become a hot spot in future research.

## Effects of the intratumoral microbiota on antitumor immunity

**Fig. 4:**
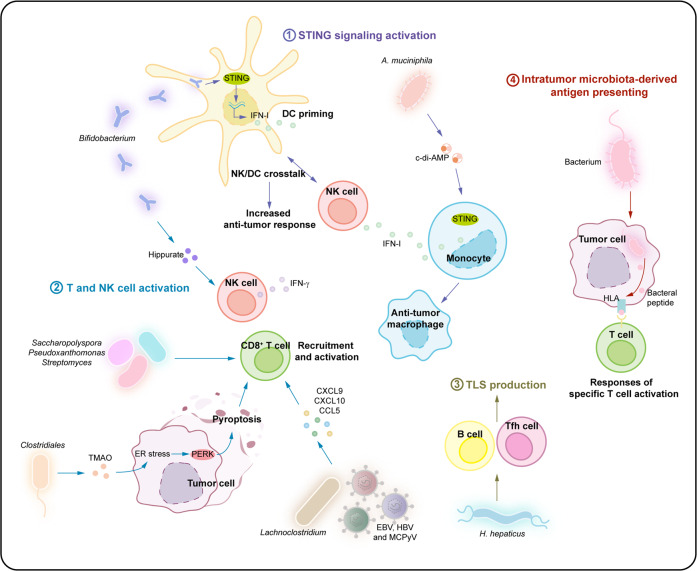
Effects of the intratumoral microbiota on enhancing antitumor immunity. The intratumoral microbiota may enhance antitumor immunity and immunotherapy efficacy via mechanisms including STING signaling activation, T and NK cell activation, TLS production, and intratumoral microbiota-derived antigen presenting. (1) STING signaling activation: The intratumoral *Bifidobacterium* can activate DCs via the STING signaling pathway. *A. muciniphila* can produce STING agonists to induce IFN-I secretion by intratumoral monocytes, further promoting macrophage reprogramming and the crosstalk between NK and DC. (2) T and NK cell activation: The intratumoral *Saccharopolyspora*, *Lachnoclostridium*, EBV, and HBV, etc. can enhance antitumor immunity by promoting CD8^+^ T cell recruitment and activation mediated by intratumoral microbiota-derived CXCL9, CXCL10 and CCL5, which further prolongs patient survival. TMAO secreted by *Clostridiales* could trigger the PERK-mediated ER stress to induce tumor cell pyroptosis, which enhances antitumor immunity mediated by CD8^+^ T cells. A high-salt diet can increase *Bifidobacterium* and intratumoral localized, leading to enhanced NK cell function and tumor regression through the elevated by-product-hippurate. (3) TLS production: The intratumoral *H. hepaticus* induces Tfh cell- and B cell-dependent antitumor immune responses, which drives the maturation of tertiary lymphoid structures. (4) Intratumoral microbiota-derived antigen presenting: Furthermore, bacterial antigens can be seized by tumor cells or DCs, which further induces the responses of tumor-specific T cells

**Fig. 5:**
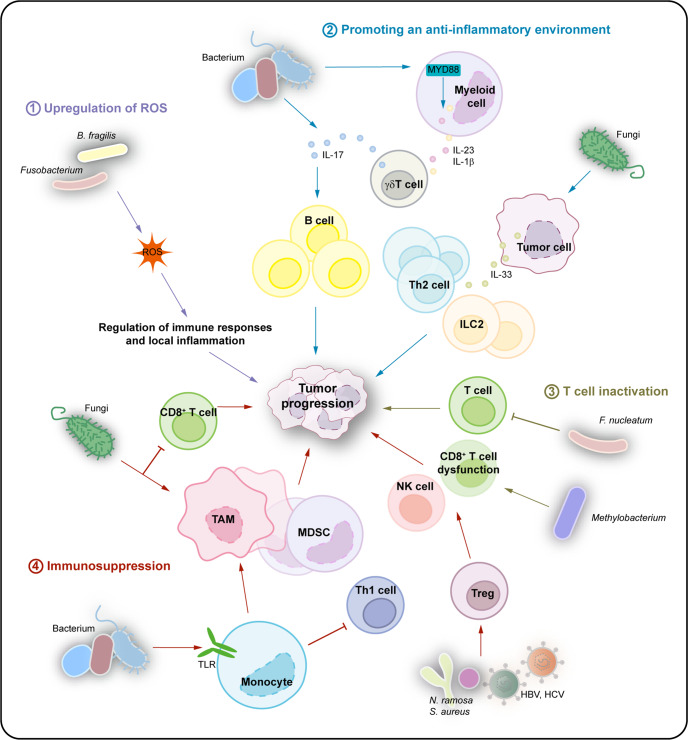
Effects of the intratumoral microbiota on decreasing antitumor immunity. The intratumoral microbiota may not only enhance antitumor immunity but also decrease antitumor immune responses and promote cancer progression through pathways including upregulation of ROS, promoting an anti-inflammatory environment, T cell inactivation, and immunosuppression. (1) Upregulation of ROS: *B. fragilis and Fusobacterium* can result in tumor progression via the production of ROS, which regulates immune responses and local inlfammation to promote tumor progression. (2) Promoting an anti-inflammatory environment: IL-17 secreted from intratumoral bacteria can promote the infiltration of intratumoral B cells that mediate tumor growth. Bacteria in tumor tissues may modulate the local anti-inflammatory tumor microenvironment by the production of IL-1β and IL-23 from myeloid cells, which leads to high levels of IL-17 derived from γδT cells, contributing to tumor progression. The fungi in tumor tissues can enhance IL-33 secretion from cancer cells to recruit Th2 and ILC2 cell infiltration, leading to tumor progression. (3) T cell inactivation: In addition, the intratumoral *F. nucleatum* and *Methylobacterium* may decrease the density of tumor-infiltrated T cells and promote T cell dysfunction in tumor tissues to induce tumor progression. (4) Immunosuppression: Lastly, intratumoral *N. ramosa*, *S. aureus*, HBV and HCV can enhance immunosuppression by Tregs to mediate cancer development. The bacteria can program TAMs via the TLR signaling pathway, increase MDSCs, and inhibit Th1 cell differentiation to mediate immune tolerance. Commensal fungi can increase TAMs and decrease T cells to inhibit the antitumor immune responses

Several studies have reported that the gut microbiota plays a key role in shaping the host immune system responses, including bidirectional roles in antitumor immunity. Recent reports have shown that the gut microbiota not only improved responses to immunotherapy by regulating antitumor immunity using checkpoint blockades,^3,47–50,89^ but also prevented antitumor immunity.^90–92^ Based on the effects of the gut microbiota on antitumor immunity, we propose that the intratumoral microbiota can shape the tumor immune microenvironment and regulate antitumor immunity, which can further affect cancer progression. Intratumoral microbiota may play distinct roles, such as in enhancing antitumor immunity and immunotherapy efficacy (Fig. 4, Table 1), or decreasing antitumor immune responses and promoting cancer progression (Fig. 5, Table 1).^7^

### Enhancing antitumor immune response

The intratumoral microbiota can enhance antitumor immunity and immunotherapy efficacy.^42,43^ The enhanced antitumor immunity can be reflected in various ways including stimulator of interferon genes (STING) signaling activation, T and natural killer (NK) cell activation, maturation of tertiary lymphoid structures, and intratumoral microbiota-derived antigen presenting (Fig. 4, Table 1).

#### STING signaling activation

The STING signaling pathway can be activated by the intratumoral microbiota (Fig. 4). *Bifidobacterium* may migrate to and colonize colon cancer sites to activate dendritic cells (DCs) via the STING signaling pathway.^93^ Systemic administration of *Bifidobacterium* induces intratumoral accumulation and alters the response to anti-CD47 immunotherapy in a STING-dependent manner. Meanwhile, local treatment with *Bifidobacterium* effectively improved the cross-priming capacity of DCs by triggering the STING signaling pathway after anti-CD47 therapy, which provided a new mechanism in which intratumoral bacteria synergize with T cell-targeted immunotherapy.^93^ In addition, *Akkermansia muciniphila*-derived STING agonists could induce the production of type I interferon (IFN-I) by intratumoral monocytes, further inducing macrophage reprogramming and the crosstalk between NK and DC, thus improving the efficacy of the ICB of melanoma patients.^44^

#### T and NK cell activation

Moreover, the tumor microbiota can shape antitumor immunity by promoting T and NK cell activation (Fig. 4). For instance, the presence of *Saccharopolyspora*, *Pseudoxanthomonas*, and *Streptomyces* in pancreatic adenocarcinoma tissues may contribute to antitumor immune responses by favoring the recruitment and activation of CD8^+^ T cells.^43^ Higher densities of CD8^+^ T and granzyme^+^ B cells were detected in long-term survival patients compared to those in short-term survival patients; no significant differences were found in macrophages, regulatory T cells (Tregs), and myeloid-derived suppressor cells (MDSCs). Furthermore, the densities of CD8^+^ T and granzyme B^+^ cells were found to have a close relationship with tumor tissues and the overall survival of pancreatic adenocarcinoma patients.^43^ Wang et al.^94^ recently reported that trimethylamine N-oxide (TMAO) secreted by the genera under *Clostridiales* could trigger the protein kinase-like ER kinase (PERK)-mediated endoplasmic reticulum (ER) stress, which enhances antitumor immunity and improve the efficacy of immunotherapy in triple-negative breast cancer induced by tumor cell pyroptosis. Intratumoral-resident microbiota, including *Lachnoclostridium*, EBV, HBV, and MCPyV could induce chemokine production and further affect CD8^+^ T cell infiltration in tumor tissues, consequently improving patient survival in cutaneous melanoma.^95–99^ The abundance of *Bifidobacterium* was increased and localized intratumorally by the induction of high-salt diet, leading to enhanced NK cell function and melanoma regression through the elevated by-product-hippurate.^100^

#### Tertiary lymphoid structure (TLS) production

In addition, intratumoral microbiota promote the maturation of TLS (Fig. 4). TLS can be formed in tumoral and peritumoral tissues due to persistent inflammation. The maturation of TLS not only facilitates lymphocyte infiltration into tumor tissues, but also enhances lymphocyte activation, which is essential for antitumor immunity. *Helicobacter hepaticus* induced T follicular helper (Tfh) cell- and B cell-dependent antitumor immune responses, which promote the development of peritumoral tertiary lymphoid structures to inhibit colon cancer growth.^101^ These results indicate that the tumor microbiota induces the production of tertiary lymphoid structures to elevate antitumor immune responses.

#### Intratumoral microbiota-derived antigen presenting

Generally, intratumoral bacteria are intracellular and can be present in both tumor and immune cells. Peptides derived from intracellular bacteria can be presented by antigen-presenting cells to further activate the responses of tumor-specific T cells (Fig. 4). A previous study observed an increase in IFN-γ-secreted melanoma-infiltrating lymphocytes exposed to different bacterial peptides, compared to control cells that were not loaded with these peptides, which indicated that peptides from intracellular bacteria presented by tumor cells elicit T cell immune reactivity and could serve as a potential target for attacking tumor cells.^42^ Moreover, in an immunofluorescence staining experiment, bacteria were present in CD45^+^ immune cells and tumor cells in the tumor microenvironment, suggesting that these cellular bacteria might improve antitumor immunity or responses to immunotherapy.^8^

### Decreasing antitumor immune response

The intratumoral microbiota may not only enhance antitumor immunity but may also exhibit an immunosuppressive effect on antitumor immunity and promote cancer progression through various mechanisms including upregulation of ROS, promoting an anti-inflammatory environment, T cell inactivation, and immunosuppression (Fig. 5, Table 1).

#### Upregulation of ROS

Commensal microbiota can produce ROS, which regulate immune responses, contributing to tumor progression (Fig. 5). Upon rupture of mucosal surface barriers, *B. fragilis* can elicit proinflammatory or immunosuppressive mechanisms to regulate immune responses in the tumor microenvironment, which results in colon cancer progression via ROS production.^5,6^ *Fusobacterium* in the gastrointestinal tract promote intestinal carcinogenesis by regulating local inflammation and the production of ROS.^90,102^

#### Promoting an anti-inflammatory environment

Furthermore, increased microbiota in tumor tissues may modulate the local anti-inflammatory tumor microenvironment to promote tumor progression (Fig. 5). IL-17 secreted from intratumoral bacteria could promote the infiltration of intratumoral B cells that mediate colon cancer growth.^56^ Neutrophils remarkably decreased the number of bacteria and tumor-associated inflammatory responses, which are essential for inhibiting colon cancer growth and progression.^56^ Moreover, commensal bacteria enhance local inflammation in lung cancer by promoting IL-17 production from γδ T cells, which results in tumor progression.^28^ Alam et al.^103^ found that the mycobiome in pancreatic ductal adenocarcinoma tissue could enhance the secretion of IL-33 from cancer cells, which further recruited T helper 2 (Th2) cells and innate lymphoid cells (ILC) 2 into the tumor microenvironment, leading to tumor progression.

#### T cell inactivation

Increasing evidence has shown that the intratumoral microbiota is negatively associated with the density of tumor-infiltrated T cells and can promote T cell dysfunction in tumor tissues (Fig. 5). *F. nucleatum* obviously decreased the accumulation of tumor-infiltrated T cells, and promoted the growth and metastasis of breast cancer.^104^ Moreover, *F. nucleatum* levels were inversely associated with CD3^+^ T cell density in breast cancer tissues,^105^ and the analysis of transcriptome and digital pathology also showed that intratumoral bacterial load was negatively correlated with T cell infiltration.^106^ Intratumoral *Methylobacterium* could induce the dysfunction of CD8^+^ tissue-resident memory cells in the tumor microenvironment of gastric cancer and promote tumor progression.^107^

#### Immunosuppression

The intratumoral microbiota has been shown to facilitate carcinogenesis by shaping an immunosuppressive tumor microenvironment (Fig. 5). Previous studies reported that the presence of the microbiota, including *Nevskia ramosa*, and *Staphylococcus aureus*, HBV and HCV in tumor tissues enhanced immunosuppression by Tregs in the tumor microenvironment to mediate prostate and liver cancer development.^108–110^ The specific microbiota containing *N. ramose*, and *S. aureus* were closely associated with the dysregulation of immune-associated genes, such as lysophosphatidylcholine acyltransferase 2, TLR3, and transforming growth factor beta-2.^108^ In mice treated with antibiotics, a decrease in bacterial load was closely correlated with reduced Tregs and enhanced T cell and NK cell activation, which induced a significant repression of melanoma B16 lung metastases.^111^ The pancreatic ductal adenocarcinoma microbiome can program tumor-associated macrophages (TAMs) via the TLR signaling pathway to mediate immune tolerance, which induces an immunosuppressive environment in pancreatic ductal adenocarcinoma.^26^ Bacterial depletion induced the reprogramming of immunity in the tumor microenvironment of pancreatic ductal adenocarcinoma, including an increase in M1-like macrophages and Th1 cell differentiation, activation of CD8^+^ T cells, and a reduction in MDSCs.^26^ Furthermore, bacterial depletion also enhanced the efficacy of immunotherapy with checkpoint inhibitors via programmed cell death protein 1 (PD-1) upregulation.^26^ Moreover, commensal fungi can increase tumor-promoting macrophages and decrease T cells by binding to Dectin-1, thus inhibiting the antitumor immune response after radiotherapy.^45^

### Condition-dependent effects

The positive and negative effects of the intratumoral microbiota on antitumor immunity are also dependent on the specific conditions of tumors, such as microsatellite instability (MSI) in colorectal carcinoma. The presence of *F. nucleatum* was negatively correlated with tumor-infiltrated lymphocytes in MSI-high colorectal cancers but was positively correlated with tumor-infiltrated lymphocytes in non-MSI-high colorectal cancers. Thus, the crosstalk between *F. nucleatum* and the immune response differs according to the MSI status of tumors, indicating that intratumoral bacteria and MSI status interact to influence antitumor immunity.^112^ Furthermore, *F. nucleatum*-enriched MSI-high colon tumors are characterized by an immunosuppressive tumor microenvironment with high levels of M2-like macrophages, which induce tumor growth and invasion.^113^ These findings indicate that *F. nucleatum* may be related to pro-tumoral immune responses in MSI-high colorectal cancers.

Therefore, it has been shown that the intratumoral microbiota affects the antitumor immune response and the efficacy of immunotherapy in many ways, which can not only play a positive role in promoting the antitumor immune response, but also can inhibit antitumor immunity. The specific effect of the forward or reverse antitumor immunity depends on the differences in the types of intratumoral flora, tumor tissue types and tumor status. Different intratumoral microbiota in different tumor tissues have distinct effects on the antitumor immune response.^8^ It is unclear exactly what factors determine or dominate the antitumor immunity effect of intratumoral microbiota. The same microbiota will have different antitumor immune responses in different tumor tissues. In the same tumor tissue, different microbiota have different antitumor immune functions. It is worth noting that there are many studies on the microbiota in pancreatic cancer, suggesting that the pancreas is an important focus of this research as it is connected to the digestive tract. Microorganisms from the outside human body and intestine colonize the pancreatic tissue through the digestive tract and mediate the antitumor immune response in pancreatic cancer. Collectively, intratumoral microbiota are involved in mediating the immune response of the tumor microenvironment and the efficacy of immunotherapy, which is also an important research direction in the future.

## Signaling pathways involved in the influence of intratumoral microbiota on tumor biology and immune response

**Fig. 6:**
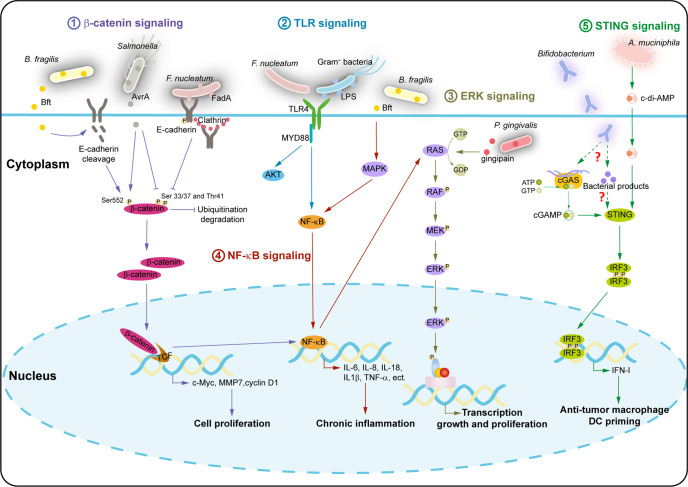
Signaling pathways involved in the influence of intratumoral microbiota on tumor biology and immune response. Intratumoral microbiota may regulate cancer cell physiology and the immune response by different signaling pathways, including β-catenin, TLR, ERK, NF-κB, and STING. (1) β-catenin signaling: The products of *Salmonella, F. nucleatum* and *B. fragilis* can directly or indirectly activate the β-catenin signaling through E-cadherin-mediated phosphorylation, thus triggering β-catenin to translocate into the nucleus and activate TCF, which stimulates downstream target gene transcription and leads to cell proliferation. (2) TLR signaling: *F. nucleatum* can bind to TLR4 on tumor cells to activate the AKT and NF-κB signaling; gram-negative bacteria-derived LPS can also be recognized by TLR4, which further triggers the NF-κB signaling pathway. (3) ERK signaling: Both *F. nucleatum*-activated NF-κB signaling pathway and gingipain produced by *P. gingivalis* can stimulate the RAS-RAF-MEK-ERK signaling cascade. (4) NF-κB signaling: Microbiota can activate NF-κB through the β-catenin or TLR signaling pathway, and Bft-mediated MAPK signaling pathways can also activate NF-κB to induce inflammatory cytokine production. (5) STING signaling: *A. muciniphila* can produce c-di-AMP to activate the STING/IRF3/IFN-I signaling pathway, which induces the polarization of anti-tumor macrophage; *Bifidobacterium* can activate the STING signaling pathway to induce DC priming either by bacterial DNA-induced cGAS recognition or other bacterial products

Above, we summarized the processes by which intratumoral microbiota regulate cancer cell physiology and the immune response. The signaling pathways involved in this regulation include ROS, β-catenin, TLR, ERK, NF-κB, and STING, among others (Figs. 3–6). Here, we discuss each signaling pathway in turn.

### ROS signaling

ROS are produced in cells catalyzed by the activity of nicotinamide adenine dinucleotide phosphate oxidase or in the redox reactions of mitochondrial respiration.^114,115^ Their roles in cancer are diverse, involving DNA damage, promoting cell proliferation, evading apoptosis and anoikis, inducing tissue invasion, and angiogenesis. ROS are also involved in one of the most recognized mechanisms of metastasis, EMT.^116^ Reportedly, *B. fragilis*-produced toxin (Bft) increased ROS levels in intestinal epithelial cells, leading to oxidization and damage to host cell DNA, resulting in malignant cell transformation.^6,76^ (Fig. 3).

Apart from the epithelium, macrophages, neutrophils, and fibroblasts can all produce ROS in the tumor microenvironment.^117^ Microbes promote high levels of ROS production in myeloid cells.^50^ Mitochondrial ROS-induced DNA damage leads to downregulation of NAD^+^, which results in the ageing of M1-like macrophages.^118^ ROS, generated by TAMs, activate matrix metalloproteinases that induce EMT programs in adjacent epithelial cells and increase tumor cell invasion.^119,120^ Additionally, MDSCs inhibit T cell function through ROS-dependent peroxynitrite production.^121^ (Fig. 5).

### β-catenin signaling

Numerous microorganisms have been identified as activators of β-catenin signaling in the gastrointestinal system, including *H. pylori*, *S. typhi*, *F. nucleatum*, and *B. fragilis*, among others, acting either directly, by secretion, or indirectly, mediated by E-cadherin.^79–83^

In particular, the effector of *Salmonella*, AvrA, is able to upregulate Wnt, Wnt receptor Frizzled 7, and T cell factor/lymphoid enhancer factor-1 (TCF/Lef1) expression.^81,122^ Further, AvrA enables β-catenin to undergo a variety of post-transcriptional modifications, all of which work together to regulate the activity of β-catenin. Phosphorylation of Ser-33/37 and Thr41 increases ubiquitin-mediated degradation of β-catenin, while phosphorylation of β-catenin at Ser-552, which results from AKT activation and can be served as a marker of proliferating stem-progenitor cells, contributes to increased nuclear β-catenin signaling.^123^ AvrA downregulates phosphorylation of Ser-33/37 and Thr41, and upregulates phosphorylation of Ser-552, thus increasing nuclear β-catenin signaling and acetylation modifications.^80,81^ Acetylation of the C terminus of β-catenin has been reported to increase its ability to activate TCF.^124^ β-catenin binds to the transcription complex TCF and is transferred to the nucleus where it is able to stimulate transcription or downstream target genes, such as c-Myc, matrix metalloproteinase-7, and cyclin D1.^80,81,125^ (Fig. 6).

Activation of β-catenin pathways by *F. nucleatum* and *B. fragilis* is mediated by E-cadherin. Rubinstein and colleagues found that *Fusobacterium* adheres to and invades epithelial and colorectal cancer cells through surface virulence factor-FadA bound to E-cadherin.^82^ Adhesion of FadA leads to phosphorylation of E-cadherin on the cell membrane and subsequently internalization of E-cadherin through clathrin. This reduces the level of phosphorylated β-catenin allowing the β-catenin accumulated in the cytoplasm to translocate into the nucleus and activate the expression of various transcription factors, such as TCF and NF-κB, and oncogenes, such as Myc and cyclin D1.^126^ At the same time, the expression of inflammatory genes, including IL-6, IL-8, and IL-18, are elevated.^82^ The *B. fragilis*-derived Bft-activated β-catenin pathway requires E-cadherin cleavage.^83^ Bft induces rapid cleavage of E-cadherin in two steps. Biologically active Bft first stimulates shedding of the E-cadherin ectodomain and then activates the host cell γ-secretase that cleaves the intracellular E-cadherin fragment.^83,127^ Proteolysis of E-cadherin promotes TCF-dependent β-catenin nuclear signaling.^128^ (Fig. 6).

### TLR signaling

TLR is a pattern recognition receptor that can recognize microbial associated molecular patterns such as lipopolysaccharides (LPS), flagellin, and peptidoglycans, among others.^129,130^ Within the TLR family, each member identifies specific pathogenic components, meaning the innate immune system is able to recognize and resist the invasion of pathogenic microorganisms.^131^ After binding to the ligand, the TLR becomes activated and signaling pathways are initiated from intracytoplasmic toll/IL-1 receptor domains, which contain several adaptors. MYD88 is a common adaptor for all TLRs and is necessary for the induction of inflammatory cytokines. Upon stimulation, MYD88 recruits a series of molecules that transmit signals and eventually activate two different signaling pathways, NF-κB and c-Jun N-terminal kinases.^131^ Numerous studies have found that TLR expression is elevated in a variety of tumor tissues.^132,133^

Recent research has demonstrated that TLR signaling plays a complex role in the effects induced by microbes within tumors. First, microbes can trigger TLR signaling in tumor cells to promote tumor progression. *F. nucleatum*, for example, binds TLR4 on colorectal cancer cells, activating NF-κB signaling resulting in an increase in inflammatory factors and downstream ERK signaling,^6,62^ and also activating AKT signaling leading to increases in the downstream metabolites 12, 13-EpOME,^85^ both of which can lead to tumor progression. LPS is the main component of the outer membrane of gram-negative bacteria and can be recognized by TLR4, meaning it activates NF-κB and increases inflammatory cytokines [IL-1β, IL-6, IL-8, tumor necrosis factor-α (TNF-α)].^79,134^ Moreover, LPS is detectable in a variety of cancers and presents a similar stained region to 16 S rRNA-fluorescence in situ hybridization.^8^ (Figs. 3 and 6).

In addition, microorganisms can bind to TLRs on immune cells to induce an inflammatory immunosuppressive environment. Jin et al. found that commensal bacteria can activate myeloid cell MYD88 to secrete IL-23 and IL-1β. These cytokines lead to increased production of IL-17 by γδ T cells, enhance local inflammation, and promote lung cancer progression.^28^ The findings of Pushalkar and colleagues adds further weight to the theory that microbes influence the tumor immune microenvironment; they found that bacteria in pancreatic ductal adenocarcinoma can bind TLRs on monocytes, polarize them into TAMs, and thereby regulate other innate and adaptive immune cells, including increasing MDSCs, inhibiting the differentiation of CD4^+^ T cells into Th1 cells, and activating CD8^+^ T cells.^26^ This combination of changes acts to construct immunosuppressive niche. (Fig. 5).

### ERK signaling

ERK signaling is one of the branches of the MAPK pathway, which is a three-tier cascade: MAPK kinase kinase (MAPKKK) activates MAPK kinase (MAPKK) which activates MAPK. ERK signaling is often interpreted as a conserved RAS-RAF-MEK-ERK signaling cascade, which is also an important tumor intrinsic signaling pathway.^135^ This series of cascading pathways begins with the transformation of inactive (GDP-bound) RAS to the active (GTP-bound) form. This change of conformation allows RAS to bind and activate MAPKKK-RAF which catalyzes the phosphorylation of the MAPKK-MEK. In turn, phosphorylated MAPKK-MEK phosphorylates and activates MAPK-ERK which then translocates to the nucleus where it activates transcription factors and promotes downstream gene expression related to growth and proliferation.^135,136^ (Fig. 6).

Microbes can activate ERK signaling in tumor cells in a direct or indirect way. The latter involves TLR signals, as mentioned above, and initiates ERK signal transduction by activating RAS.^62^ Alternatively, microbial products within tumor cells may directly activate upstream signals.^86^ (Figs. 3 and 6).

### NF-κB signaling

Tumor-promoting inflammation is one of the defining characteristics of cancer.^137^ NF-κB signaling plays a key role in chronic inflammation caused by microbes. As mentioned above, microorganisms can activate NF-κB through β-catenin and TLR signaling, and this activation leads to the release of a variety of inflammatory factors and induces chronic inflammation. In addition, this process may occur in reverse, with chronic inflammation recruiting immune cells which release inflammatory mediators, which in turn activate NF-κB. Together, these processes form a positive feedback loop, further promoting tumor progression.^138^ Kostic et al. confirmed that *Fusobacterium* is enriched in colorectal adenomas and carcinoma where it can increase the number of CD11b^+^ myeloid cells and level of inflammatory markers in tumors. This suggests that *F. nucleatum* modulates the immune microenvironment and promotes tumor progression by inducing NF-κB-driven inflammation in early tumorigenesis.^41,126^ In addition, many studies have shown that enterotoxin (Bft) activates multiple MAPK pathways (via p38, ERK and JNK) in intestinal epithelial cells, which further activates NF-κB signaling and induces the mucosal inflammatory response.^83,139–142^ (Fig. 6).

### STING signaling

STING is a cytosolic DNA sensing protein that is activated in combination with cyclic dinucleotides and can induce expression of IFN-β and other pro-inflammatory genes.^143^ Cyclic dinucleotides are second messenger in bacteria, including cyclic di-GMP, cyclic di-AMP (c-di-AMP), 2'3’-cyclic GMP-AMP (cGAMP).^144^ In the case of pathogen infection, cyclic GMP-AMP synthase (cGAS) can directly recognize pathogen-derived DNA, catalyse the synthesis of ATP and GTP into cGAMP, and activate the downstream STING/tank-binding kinase 1/interferon regulatory factor 3 (IRF3)/IFN-β signaling pathway in host cells.^145,146^ STING expression is found in macrophages, DCs, and lymphocytes, as well as endothelial and epithelial cells.^147,148^ Studies have shown that the cGAS/STING signaling pathway can strongly regulate macrophage polarization to the M1-like phenotype and exert a powerful anti-tumor effect.^147,149^ In several tumor models, bacteria (e.g., *A. muciniphila*) can produce STING agonists (such as c-di-AMP) which target monocytes and induce cell polarization in the direction of anti-tumor macrophage. This also promotes NK cell activation and crosstalk with DCs through the production of IFN-I.^44^ In addition, *Bifidobacterium* can directly activate the STING signal of DCs, resulting in DC priming.^93^ However, it is unclear whether the mechanism of STING activation is caused by bacterial DNA-induced cGAS recognition or other bacterial products. IFN-I produced by STING pathway activation in macrophages also activates tumor-infiltrating Batf3 DCs and tumor antigen-specific CD8^+^ T cells.^147,150^ Similar findings have been reported in relation to gut microbiomes, where oral administration of *Lactobacillus rhamnosus* GG, in combination with ICB, shifts the microbial community towards the enrichment of *Lactobacillus murine* and *Bacteroides uniformis*, thus inducing cGAS/STING-dependent IFN-I production which results in DC activation and CD8^+^ T cell recruitment in tumors.^151^ (Figs. 4 and 6).

### Other signaling (complement, RhoA/ROCK, PERK)

In addition to the signaling pathways discussed so far, which involve interactions between multiple aspects of different pathways, tumor microorganisms may also activate other, more specific, signaling pathways.

Mannose-binding lectin (MBL), as reported by Aykut et al., recognizes fungal glycan and promotes activation of the complement cascade, thus mediating tumor progression via complement receptors on tumor cells.^27^ In tumor cells, C3aR-, C5aR1-, or MAC-mediated complement signaling triggers the activation of the PI3K-AKT pathway, ultimately promoting cell proliferation.^152,153^ The complement system can also promote tumor cell motility and aggressiveness by stimulating production of metalloproteins by tumor cells, inducing degradation of the extracellular matrix, and increasing stress fibers and filopodia.^153–155^

Bacteria in tumor cells can inhibit the RhoA/ROCK signaling pathway, resulting in adaptation of circulating tumor cells to fluid shear stress by adjusting the cytoskeleton and promoting distant colonization.^87^ In ovarian cancer, it has been found that TAGLN can induce tumor progression by upregulating the RhoA/ROCK pathway in tumor cells when stimulated by environmental stiffness.^156^

In addition, bacterial metabolites can also activate PERK-mediated endoplasmic reticulum stress in tumor cells, thereby promoting gasdermin E-mediated tumor cell pyroptosis.^94^ The double-stranded RNA-dependent PERK is a key transmembrane protein that regulates endoplasmic reticulum stress.^157^ PERK can promote malignant cell proliferation and tumor angiogenesis.^158–160^ Extracellular stress, intracellular stress, and oncogene activation can activate PERK.^161^ As mentioned above, microorganisms can activate β-catenin signaling to increase c-Myc expression.^80–82,125^ Amplified levels of c-Myc can significantly upregulate transcription and translation in the endoplasmic reticulum, resulting in the accumulation of numerous unfolded proteins, which in turn activates the PERK signaling pathway.^161^ This may explain why microbes induce endoplasmic reticulum stress.

## Effects of the intratumoral microbiota on anti-cancer therapy

At present, the most commonly used antitumor therapies are radiotherapy, chemotherapy and immunotherapy. What is the impact of the intratumoral microbiota on these treatments? The relationship between intratumoral microorganisms and immunotherapy has been elaborated above in detail. A simple example is that the combination of oral *Megasphaera sp.XA511* and anti-PD-1 treatment was found to significantly inhibit tumor growth in the 4T1 tumor-bearing mouse model. However, whether the impact of *Megasphaera sp.XA511* is due to it reaching the tumor site has not been explained.^162^

In terms of chemotherapy and radiotherapy, current research mainly focuses on how the intratumoral microbiota leads to poor efficacy, such as drug resistance and relapse after treatment. The tumor microenvironment not only affects the efficacy of antitumor therapy, but also plays a key role in inducing resistance to antitumor drugs. In addition to cancer cells, other cell types (immune cells, stromal cells, etc.) can influence the development of drug resistance.^163,164^ With in-depth research, the influence of intratumoral microbiota on the form of resistance to antitumor therapy has been gradually elucidated.^165^ Antitumor therapy combined with intratumoral microbiota may provide new avenues for reducing the occurrence of drug resistance in the future. *Gammaproteobacteria* expresses cytidine deaminase, which can completely metabolize gemcitabine, thereby enhancing drug resistance.^23,166^ Meanwhile, Geller et al. reported that 86 (76%) human pancreatic ductal adenocarcinoma samples were positive for bacteria, mainly *Gammaproteobacteria*, which might contribute to gemcitabine resistance in pancreatic ductal adenocarcinoma.^23^ In addition, increased levels of the oral pathogens *Aggregatibacter actinomycetemcomitans* and *P. gingivalis* were observed in pancreatic cancer patients, which promoted the expression of cytidine deaminase, and impacted the occurrence of chemoresistance.^167^ In the mouse colorectal cancer model CT26, intratumoral administration of *E. coli* not only affected the activity and concentration of gemcitabine at the tumor site, but also affected the development of drug resistance.^168,169^ In patients with colorectal cancer, another important intratumoral bacteria, *F. nucleatum* has also been shown to promote the development of oxaliplatin resistance during treatment, by activating the innate immune system and inducing autophagy.^170,171^ Biliary stent placement and chemotherapy drugs (gemcitabine and paclitaxel) may partially promote the growth of *Enterobacteriaceae*, which are considered to enhance resistance to chemotherapy.^172^ The relationship between the occurrence of chemotherapy resistance and tumor microbiota has been widely reported, but studies on radiotherapy are still lacking. Oral administration of vancomycin-sensitive bacteria, *Lachnospiraceae*, leads to elevated butyric acid levels in the whole body and tumor sites, thus reducing the efficacy of ionizing radiation.^173^

In addition to drug resistance, there are few studies on the relationship between intratumoral microbiota and tumor recurrence. Studies have shown that the increase of the relative abundance of Operational Taxonomic Unit_104, is directly related to the recurrence of colon cancer.^174^ Oral squamous cell carcinoma is closely associated with changes in oral microbiota, especially *F. nucleatum*. It was found that patients who were positive for *F. nucleatum* had a lower recurrence rate, reduced metastatic recurrence, and significantly longer metastasis-free survival.^175^ With the further research of intratumoral microbiota in chemoradiotherapy resistance and recurrence, targeting the microbiome could provide a new adjuvant strategy for antitumor therapy.

## Application of the microbiota in cancer therapy

Given the various mechanisms underlying tumor regression caused by local microbial infection, the microbiota is used for the treatment of cancers (Tables 2–5). The microbiota can be engineered to improve and enhance its antitumor effects based on microbiota-intrinsic mechanisms. In addition, the intratumoral microbiota can induce innate and adaptive immune responses to prevent tumor progression.^176–184^ At present, several microbiota and their related preparations have been approved by the Food and Drug Administration (FDA) for the treatment of cancer patients (Table 6).^14,185,186^ These drugs can directly kill tumor cells after reaching the tumor sites, or play an immunomodulatory role in enhancing antitumor immunity.

**Table 2:** Therapeutic strategies using microbiota as vectors for cancer therapy

Classification	Therapeutic strategy	Microbiota type	Tumor type	Effect	Ref
Vector producing cytotoxic drugs	Incubated with radiolabeled antibody	*Listeria*	Pancreatic cancer	Decreased metastasis	^191^
	Engineered with NO	*E. coli*	Breast cancer	Tumor regression	^192^
Vector for gene delivery	Bacteria-based DNA delivery system	*Listeria*	Breast cancer	The potential for in vivo gene delivery and gene therapy of cancers	^193^
	FASL expression	*Salmonella*	Breast and colon cancer, melanoma	Evident antitumor responses	^194^
	Cytotoxic Cp53 peptide expression	*Salmonella*	Cervical and breast cancer	Induced the killing of tumor cells	^195^

**Table 3:** Regulation of the immune responses by the microbiota used for cancer therapy

Classification	Therapeutic strategy	Microbiota type	Tumor type	Effect	Ref
Probiotics	NA	Probiotic bacteria	Colon cancer	Epigenetic alterations	NCT03072641
		DTA81	Colon cancer	Inhibition of cancer development	^197^
Microbiota without engineering	NA	*E. coli* strain MG1655	Colon, renal, and prostate cancer	High production of TNF-α	^198^
		*H. hepaticus*	Colon cancer	Promotion of Tfh-associated antitumor immune responses	^101^
Engineered microbiota expressing tumor antigens	Tumor vaccine	*Listeria* and *Salmonella*	Solid tumor	Delivery of tumor antigens to the immune system	^199,200^
	Express immunodominant T cell antigens to infect tumor cells	Tetanus toxoid, poliovirus, or measles virus	Solid tumor	Induction of memory T cell responses	^190^
Engineered microbiota expressing immunoregulatory factors	Bacteria-mediated DNA delivery	Non-pathogenic bacteria	Ovarian and colon cancer	Recruitment of more phagocytic cells, enhancement of inflammatory responses	^187^
	*Salomonella* expressing β-galactosidase	*S. typhimurium*	NA	Induction of substantially stronger immune responses	^202^
	*S. typhimurium* expressing of FlaB	*S. typhimurium*	Colon cancer	Reduction of tumor progression, polarization of TAMs to M1-like macrophages, increased proinflammatory cytokine production	^188^
	*Salmonella* expressing IL-2	*Salmonella*	Colon cancer with liver metastasis	Enhancement of antitumor immunity in a NK and CD8^+^ T cell-depended way	^203,204^
	*Salmonella* expressing LIGHT	*Salmonella*	Breast, colon, and lung cancer	Induction of CD4^+^ and CD8^+^ T cell responses	^205^
	*Listeria* with α-galactosylceramide	*Listeria*	Breast cancer	Stimulation of NKT cells, inhibition of tumor progression	^206^
Microbiota combined with checkpoint inhibitors	*Bifidobacterium* + anti-PD-L1 antibody	*Bifidobacterium*	Breast cancer	Inhibition of tumor growth, enhancement of cancer immunotherapy	^210^
	Talimogene laherparepvec (Tvec) + pembrolizumab	Tvec	Advanced melanoma	Objective response rate was 62%, reprogramming of immunogenic microenvironment	^209^
	Microbiota engineered with secreting checkpoint inhibitors	*C. novyi-NT* and *C. sporogenes*	Solid cancer	Immune activation of T cells	^211^

**Table 4:** Intratumoral injection of *C. novyi-NT* for cancer therapy

Classification	Therapeutic strategy	Phase	Tumor type	Effect	Ref
Pre-clinical	Intratumoral injection	NA	Spontaneous solid tumors	Well tolerated, objective response was 37.5%	^212^
	Branched gold nanoparticle-coated spores		Prostate cancer	Significant antitumor response	^213^
Clinical treatment/trial	Intratumoral injection	I	Injectable, treatment-refractory solid tumors	Significant but manageable toxicity, tumor-confined cell lysis, and enhance systemic tumor-specific T-cell responses	NCT01924689^214^
		I	Treatment-refractory solid tumor malignancies	No results posted	NCT00358397
		I	Treatment-refractory solid tumor malignancies	No results posted	NCT01118819
		I	Advanced leiomyosarcoma	Reduction of tumor burden, killed and necrotized tumor cells	NCT01924689^212^
		I	Treatment-refractory solid tumors	No results posted	NCT03435952

**Table 5:** Oncolytic viruse for antitumor immunotherapy in clinical trials

Virus	Name	Modification	Tumor type	Phase	Effect	Identifier
Adenovirus	DNX-2401	Δ24-RGD insertion	Glioblastoma	Done	Enhancing immune response and prolonging patient survival	NCT00805376
	H101	E1B deletion, partial E3 deletion	Hepatocellular carcinoma, Pancreatic ductal adenocarcinoma	III/I	No results posted	NCT03780049 NCT05303090
	Enadenotucirev (ColoAd1)	Chimeric Ad11/3 group B	Resectable colon, bladder, Non-small cell lung cancer, renal cell carcinoma	I	Driving an immune response and tolerating enadenotucirev	NCT02053220
	CG0070	E3 deletion; GM-CSF insertion	None muscular invasive bladder cancer	III	No results posted	NCT04452591
Herpesvirus	T-VEC	ICP34.5 deletion, US11 deletion, GM-CSF insertion	Melanoma, soft tissue sarcoma	II	No results posted	NCT03555032 NCT04599062
	HF10	UL56 deletion, selected for single copy of UL52	Refractory head and neck cancer, squamous cell carcinoma, skin Carcinoma of the breast, malignant melanoma	I	No results posted	NCT01017185 NCT02428036
	HSV-1716	ICP34.5 deletion	Malignant pleural mesothelioma	II	No results posted	NCT01721018
Vaccinia virus	PexaVec (JX594)	GM-CSF insertion, TK disruption	Primary or metastatic hepatic carcinoma	I	No regrowth of the tumor site occurred in all patients	NCT00629759
	rVV-740CTA	Cancer testis antigen (CTA) epitopes, CD80 and CD154(40 L) expression	Breast carcinoma	I	No results posted	NCT03110445
	GL-ONC1	TK disruption, haemagglutin disruption, F14.5 L disruption	Peritoneal carcinomatosis	II	Lysis of tumor cells and weak adverse reactions	NCT01443260
Measles virus	MV-NIS	Edmonston vaccine measles strain; insertion of sodium-iodide symporter	Recurrent ovarian, primary peritoneal or fallopian tube cancer	II	No results posted	NCT02068794
Reovirus	Reolysin	None	Metastatic breast cancer	II	Significantly longer overall survival in the combination tharapy	NCT01656538
Coxsackie virus	Cavatak	Wild -type coxsackievirus A21	Non-muscle invasive bladder cancer	I	Tumor cell death, enhancing immune responses	NCT02316171

**Table 6:** FDA approved drugs/therapies regarding the microbiota in cancer therapy

Drug	Experimental drug	Target	Associated Microbial	Tumor type	Effect	Ref
ADI-PEG 20	Pegargiminase	None	Mycoplasma	Hepatocellular carcinoma	Killing the melanoma cells by activating caspase 8/10-dependent apoptotic pathways	^185^
BCG	BCG vaccine (Nippon BCG Seizo KK)	None	*Mycobacterium bovis*	Non Muscle Invasive Bladder Cancer (NMIBC)	Activation of TLR2 and TLR4	^186^
Talimogene laherparepvec (T-VEC)	T-VEC	CSF	Herpes simplex virus-1 (HSV-1)	Advanced melanoma	Triggering cancer cell lysis	^14^
L-asparaginase	L-asparaginase	ASN	*E. coli*	Acute lymphoblastic leukemia	Degrading asparagine and glutamine	^185^

### Vectors for cancer therapy

The microbiota organisms can serve as vectors to deliver antitumor agents for cancer therapy (Table 2). Furthermore, based on the unique ability of the microbiota to selectively grow in tumor tissues, a therapeutic strategy was developed to administer engineered microbiota, which subsequently colonize the tumor tissues.^187–189^

The most effective way to strengthen antitumor effect is to engineer a bacteria vector expressing cytotoxic drugs.^190^ According to clinical needs, bacteria vectors can be engineered or reprogrammed to deliver and produce antitumor agents. Live tumor-targeting bacteria can be applied as a monotherapy approach or in combination with other antitumor strategies.^190^ A previous study incubated a radiolabeled antibody with an attenuated *Listeria* strain and observed that, after administration, the number of metastases in a mouse model decreased.^191^ Another study focused on an engineered *E. coli* system, leading to the production of nitric oxide and further tumor regression.^192^

In addition, the microbiota serves as a vector for gene delivery into tumor cells. A study on a bacteria-based DNA delivery system revealed the potential of *Listeria monocytogenes* for in vivo gene delivery and therapy in cancers.^193^ *Salmonella* strains expressing the Fas ligand showed evident antitumor responses in a Fas-dependent way.^194^ Another study reported that cytotoxic Cp53 peptide expression induced the killing of tumor cells via bacterial autolysis to release Cp53.^195^

### Regulation of the immune responses against tumors

Except for the antitumor effects via the delivery of antitumor agents or genes, the microbiota can induce innate immune responses and adaptive immunity against tumor cells (Table 3).^177,178,180,181,184,190^

The gut microbiota can be regulated by probiotics, which can affect inflammation responses and the immune system, and are helpful for antitumor immunity (Table 3).^196^ In a clinical trial (NCT03072641), the effects of probiotic bacteria on colon cancer-related microbiota alterations were investigated. The probiotic candidate DTA81 administrated orally may inhibit colon cancer development.^197^

Even without engineering, the microbiota may enhance antitumor immunity (Table 3). The non-pathogenic *E. coli* strain MG1655 was observed to target tumor cells due to the high production of TNF-α from *E. coli* strains, specifically within tumors, which may provide a potential platform for tumor therapy.^198^ Furthermore, immunogenic intestinal bacteria can induce Tfh-related antitumor immune responses in colon cancer tissues, demonstrating a potential therapeutic method for colorectal cancer.^101^

Engineered bacteria can activate and enhance antitumor immunity by expressing either tumor antigens or immune-related factors (Table 3). Several studies have shown that live microbiota can be used as vectors expressing tumor antigens for tumor vaccination.^199–201^ Furthermore, the microbiota can express immunodominant T cell antigens to infect tumor cells, as well as present the antigens in tumor cells, leading to the induction of memory T cell responses.^190^ During childhood, the immune system recognizes these antigens and generates memory T cells. On recognition of these antigens in tumor cells, memory T cells can be stimulated, leading to the killing of infected tumor cells. Additionally, antigens may spread from destroyed tumor cells, which may mediate antitumor immunity in uninfected tumor cells.

Furthermore, an engineered microbiota expressing immunoregulatory factors could induce and activate immune responses (Table 3). Bacteria-mediated DNA delivery into phagocytic cells was reported to be possible, and the vector recruited more phagocytic cells into the tumor tissues, which enhanced the inflammatory responses of antitumor effects.^187^ A previous study showed that *Salmonella* has been used as a delivery vehicle for the expression of β-galactosidase, which induces substantially stronger immune responses.^202^ Zheng et al.^188^ used an engineered *Salmonella typhimurium* strain to produce *Vibrio vulnificus* flagellin B to guide cancer immunotherapy. This engineered flagellin B-secreting bacterium effectively reduced tumor progression based on TLR5 and ligand signaling pathway-mediated immune responses in the tumor microenvironment, such as the polarization of TAMs to M1-like macrophages, further increasing the secretion of pro-inflammatory cytokines, such as IL-1β and TNF-α.^188^ Moreover, *Salmonella* strains expressing IL-2 may enhance antitumor immunity in an NK- and CD8^+^ T cell-dependent manner.^203,204^ In addition, *Salmonella* strains can be engineered to express homologous to lymphotoxin, a TNF superfamily member, which has been investigated in CD4^+^ and CD8^+^ T cell responses in subcutaneous and metastatic tumor tissues in a mouse model.^205^ Moreover, *Listeria* harboring α-galactosylceramide was used in cancer therapy, and results showed that this engineered bacterium could stimulate NKT cells, further inhibiting tumor progression and prolonging survival in a mouse tumor model.^206^

Recent evidence has shown that microbiota combined with checkpoint inhibitors has been used in cancer therapy in preclinical and clinical studies (Table 3).^207–209^ Oral administration of *Bifidobacterium* combined with anti-PD-L1 antibody therapy almost completely inhibited tumor growth, indicating that the microbiota may regulate and enhance cancer immunotherapy.^210^ Patients with advanced melanoma treated with talimogene laherparepvec combined with pembrolizumab showed an objective response rate of 62%.^209^ These results indicate that intratumoral microbiota infection could reprogram the immunogenic microenvironment, which renders the tumors sensitive to immune checkpoint inhibitors. Furthermore, microbiota engineered with secretory checkpoint inhibitors could be a promising therapeutic approach for cancer therapy. Such checkpoint inhibitors may include a soluble PD-1 extracellular domain or an anti-PD-1 antibody, which could bind to PD-L1 on tumor or other antigen-presenting cells and further activate T cells.^211^

### Intratumoral injection of Clostridium novyi-NT

Increasing evidence has shown that intratumoral injection of *C. novyi-NT* precisely eliminated tumor tissues (Table 4). Roberts et al.^212^ reported that *C. novyi-NT* injection in spontaneous solid tumors was well tolerated; among 16 dogs, six had objective responses (37.5%), of which three had complete and three had partial responses. In addition, branched gold nanoparticle-coated *C. novyi-NT* spores have been designed for tumor therapy and were successfully injected into tumor tissues under the guidance of computed tomography, and a significant antitumor response was observed in a prostate tumor-bearing mouse model.^213^

Some phase I clinical trials investigated microbial colonization in a large number of patients treated with intratumorally-administered *C. novyi-NT* spores.^214–218^ Among these clinical trials, one study reported that the tumor burden in treatment-refractory advanced solid tumor patients was reduced after local treatment with *C. novyi-NT*, and tumor-specific T cell responses were enhanced.^214^ Moreover, *C. noyvi-NT* spores reduced the tumor burden in one patient with advanced leiomyosarcoma.^212^ Meanwhile, the clinical characteristics required to kill tumor cells were found and evaluated by computed tomography in a metastatic shoulder lesion after injection with *C. novyi-NT* spores.^212,214^ The results of the lesion biopsies indicated that the tumor cells were extensively killed and necrotized. However, oncolytic bacterial therapy alone failed to eliminate all tumor cells, leading to tumor progression. Therefore, all these results demonstrate that further clinical trials will be warranted in cancer patients.

### Oncolytic viruses

In addition, the study of oncolytic viruses in antitumor therapies has also achieved charming results (Table 5). Oncolytic viruses exert their antitumor effects by utilizing the sensitivity of tumor cells to viral infection, taking advantage of the dysregulated pathways to induce cell lysis. At the same time, the antigens released after tumor cell lysis can further activate antitumor immunity. Besides, oncolytic viruses can also destroy blood vessels and thus reduce tumors.^219,220^ The disadvantage of oncolytic viruses is that they only target specific tumors, lack tumor specificity, and may cause large untargeted replication and toxicity. Attention should be paid to safety during clinical use.^221^ In 2015, the FDA approved a second-generation oncolytic herpes simplex virus type 1 (HSV-1) talimogene laherparepvec (T-VEC or Imlygic) containing granulocyte-macrophage colony-stimulating factor (GM-CSF) for the treatment of metastatic melanoma, which is the only oncolytic virus immunotherapy approved by the FDA, the remaining oncolytic viruses in clinical trials cover almost all solid tumors.^220,222^ In a phase Ib trial, Coxsackie virus A21, a virus that naturally targets intercellular cell adhesion molecule-1, was well tolerated in combination with pembrolizumab and partially upregulated the number of PD-L1^+^ tumor cells.^222^ DNX-2401, known as Delta-24-RGD, is an adenovirus designed to selectively replicate in Rb deficient cells. A phase I trial showed that 20% of patients with recurrent malignant gliomas showed a favorable clinical response after intratumoral injection of DNX-2401.^223^ At present, the most common viruses in clinical trials using oncolytic viruses to treat tumors are adenovirus, HSV-1 and poxviruses, which reflect a deeper understanding of DNA viruses.^224^ Furthermore, reovirus belongs to the family Reoviridae, a non-enveloped double-stranded RNA virus, which is an attenuated Reovirus type 3 Dearing strain and has been widely studied as an antitumor agent.^225,226^

Studies have shown that oncolytic viruses show better efficacy when combined with classical clinical antitumor therapies. MeV in combination with a common chemotherapeutic agent, such as gemcitabine, promotes lysis of senescent cancer cells in a variety of tumors.^227,228^ In a phase III trial, the combination of PD-1/PD-L1 and TG4010, a modified vaccinia virus Ankara, significantly improved treatment outcomes in patients with advanced non-small cell lung cancer.^229,230^ Nishio et al. demonstrated that in a xenograft human neuroblastoma mouse model, chimeric antigen receptor T cells combined with AdV, arming the chemokine RANTES and the cytokine IL-15, can specifically enhance the migration and proliferation of chimeric antigen receptor T cells and improve the survival of patients.^231^

## Targeting the microbiota for cancer therapy

The application of microbiota in the treatment of tumors has achieved good efficacy, however some microbiota in the tumor sites promote the formation of an immunosuppressive microenvironment, leading to the development of treatment resistance and other effects that inhibit antitumor efficacy. Can we target these intratumoral microbiota to produce favorable results?

The microbiota provide, not only a therapeutic strategy for cancer treatments, but can also be targeted for cancer therapy. Based on the regulatory relationship between the gut microbiota and the immune system, depletion of the gut microbiota has been studied in cancer treatment, especially in colon cancer. In colon and melanoma cancer models, antibiotic therapy in mice compromised the efficacy of immunotherapy using anti-IL-10/CpG oligodeoxynucleotides.^5^ This antibiotic therapy led to a decrease in the gut microbiota and a decline in inflammatory cytokine production.^5^ In a clinical trial (NCT04660123), gastric cancer patients were administrated with bismuth colloidal pectin granule quadruple therapy to deplete *H. pylori*, and the adverse effect incidence and symptom improvement were investigated.^232^ Furthermore, itraconazole therapy for several cancers has been evaluated in preclinical experiments and clinical trials (NCT02749513).^210,233,234^

The use of antibiotics in cancer patients can lead to the suppression of gut microbiota, resulting in the weakening of the antitumor immune response. However, some studies have shown that in pancreatic cancer, the expression of PD-1 is also up-regulated while the intratumoral microbiota is eliminated, which promotes the immune response to a certain extent.^26,235^ Increasing clinical data suggest that systemic use of antibiotics can lead to less effective immune checkpoint inhibitors.^235,236^ In addition, several studies in patients with lung, colon, and pancreatic cancer have shown that removal of the tumor microbiota can enhance the inflammatory process of the tumor, inhibit tumor growth, or alter tolerance to immunogenic tumor microenvironment, thus improving the outcome of antitumor treatment.^25,28,111^ These seemingly contradictory results suggest that we need to fully understand the complexity of the tumor microenvironment and the contradictory microbiota in the treatment process when targeting microbiota to improve the outcome of antitumor immunity. Whether microbiota are agonists or inhibitors in the treatment of cancer depends on our therapeutic strategy and requires further in-depth discussion of the mechanisms involved.^237^ As antibiotic use may lead to the dysregulation of microbiota and the development of drug resistance, bacteriophages, as a more precise biologic agent targeting intratumoral microbiota, are also being studied. Bacteriophages can eliminate specific bacteria and are, therefore, more suitable for clinical use by targeting the tumor microbiome as well as modulating immunity.^238^

## The diagnostic and prognostic roles of intratumoral microbiota

Due to the abundance of microorganisms in tumors and the presence of tumor type- and subtype-specific microbial profiles, the intratumoral microbiota has the potential to be used as a diagnostic tool (Table 7). Increasing articles have reported the role of intratumoral microorganisms in diagnosis.^90,239,240^ Large data analyses have shown that the intratumoral microbiome characteristics of head and neck squamous cell carcinoma are related to the clinicopathological features of the tumor (including tumor stage and histological grade).^241^ Mycoomic studies have shown a clear association between specific fungi and patient age, tumor subtype, smoking status, and response to immunotherapy. But whether these fungi are strongly or causally related remains to be determined.^30^

**Table 7:** Intratumor microbiota as a diagnostic/prognostic marker in cancer patients

Diagnostic/prognostic marker	Microbiota	Tumor type	Clinical role	Associated clinicopathological characteristics*	Ref
Diagnosis	*Fusobacterium*,*Treponema*	Head and neck squamous cell carcinoma	Beneficial to evaluate tumor stage and histological grade	Host-gender, host-age, tumor stage, neoplasm histologic grade	^241^
	*Malassezia*	Pancreatic cancer	Stratifying patient survival	Age, tumor subtypes, smoking status	^30^
	*P. gingivalis*, *A. actinomycetemcomitans*	Pancreatic cancer	Aetiology of pancreatic cancer	Age, race, sex, smoking status, alcohol consumption, BMI, history of diabetes	^244^
Prognosis	*H. pylori*	Gastric cancer and colorectal adenoma	Predicting disease risk and progression	Not mentioned	^243^
	*Haemophilus parainfluenzae, S. marcescens, Acinetobacter jungii, Streptococcus constellation*	Non-small cell lung cancer	Predicting two-year survival	Malignancy, EGFR mutation, first-line outcome, survival	^247^
	*Frankia sp., Trueperella pyogenes*	Papillary thyroid carcinoma	Better prognosis	MACIS (distant metastasis, patient age, completeness of resection, local invasion, and tumor size) score, clinical variables	^248^
	*Corynebacterium, Staphylococcus*	Nasopharyngeal carcinoma	Shortening disease-free survival and distant metastasis-free survival	Not mentioned	^249^
	*F. nucleatum*	Esophageal squamous cell carcinoma	Poor prognosis, recurrence	Not mentioned	^245^
		Colorectal cancer	Independently good prognostic factor	Tumor location, microsatellite instability status	^250^
		Anal squamous cell carcinoma	Prolonging overall survival and disease-free survival	Age, sex, tumor invasion depth,TNM stage, tumor grade, lymphovascular, perineural invasions	^251^

*When analyzing intratumor microbiota as diagnostic and prognostic markers, other clinicopathological factors (such as age and tumor subtype) often cannot be excluded, which may also affect the diagnostic and prognostic analysis.

In addition, research has shown that the tumor microbiome correlates with survival rates across different patients, making it a potential prognostic tool (Table 7). Some intratumoral microbiota may be closely associated with poor prognosis of patients with tumors. *H. pylori* in gastric cancer and colorectal adenoma contributed to a higher disease risk and worse disease conditions, which were positively associated with the protein CagA levels.^242,243^ Another study showed that *F. nucleatum* and *P. gingivalis* were correlated with a higher risk of pancreatic cancer.^244^ In addition, elevated levels of *F. nucleatum* were closely correlated with advanced stage and tumor recurrence of esophageal squamous cell carcinoma.^245^ Tumor patients with high levels of intratumoral bacteria had poor recurrence-free survival and worse clinical response to neoadjuvant chemotherapy.^245^ Analysis of The Cancer Genome Atlas data showed that *Proteobacteria* was a major contributor to the poor prognosis of pancreatic duct adenocarcinoma.^246^ Logistic regression analysis of four bacteria in first-line treated non-small cell lung cancer samples can effectively predict the 2-year survival rate of patients.^247^ Studies of papillary thyroid carcinoma have also shown that the intratumoral microbiota predicts the prognosis of patients with different sexes and subtypes.^248^ Qiao et al. conducted a retrospective cohort study of biopsy specimens from 802 patients with nasopharyngeal carcinoma in two hospitals of China, and found that the intratumoral microorganisms were mainly *Corynebacterium* and *Staphylococcus*, which could be used as prognostic tools, and the high bacterial load was negatively correlated with disease-free survival, survival without distant metastasis, overall survival and T cell infiltration.^249^ High levels of *F. nucleatum* in esophageal squamous cell carcinoma showed poor response to neoadjuvant chemotherapy and predicted worse recurrence-free survival.^245^ However, in patients with stage II/III non-MSI-high/non-sigmoid colorectal cancer receiving oxaliplatin-based adjuvant therapy, intratumoral *F. nucleatum* is an independently good prognostic factor.^250^ Similarly, for anal squamous cell carcinoma patients undergoing abdominal perineal resection after radiotherapy/chemoradiotherapy with high levels of *F. nucleatum* had longer overall survival and disease-free survival.^251^ Thus, intratumoral microbiota can be used as biomarkers for diagnosis and prognosis to provide potential treatment guidance for patients with different risk stratifications.

Until now, most research on intratumoral microbes has been based on surgically removed samples, but not all cases present the opportunity to operate. Recent studies have shown that microorganisms in non-surgically obtained samples can also be used for research and diagnosis. A study compared the microbiome of pancreatic duct adenocarcinoma samples obtained using endoscopic ultrasound-guided fine-needle aspiration biopsy with those obtained by surgical resection, and the results showed that microorganisms obtained by biopsy were contained within surgically removed samples.^252^ The organisms obtained from non-small cell lung cancer broncho alveolar lavage fluid are clearly correlated with intratumoral microbiota, and their classification and abundance can be used to assess the severity of non-small cell lung cancer.^253^ *Proteobacteria* is significantly enriched in the broncho alveolar lavage fluid of non-small cell lung cancer, and further subdivision of the bacterial communities is associated with adenocarcinoma and squamous cell carcinoma respectively.^254^ In addition, there is growing evidence to prove that tumor-associated circulating microbial DNA is a potential biomarker in cancer liquid biopsies,^255^ and this approach will be more valuable given the potential for microbial transport into tumors through cancer cells or immune cells. In the future, the use of simpler and non-invasive methods to detect intratumoral microorganisms may be very clinically promising.

## Conclusions and perspectives

In conclusion, intratumoral microbiota have diverse sources, organ composition and tissue distribution, and may be inextricably related to gut microbiota. Intratumoral microbiota play an important role in the regulation of tumor progression and therapeutic efficacy. Moreover, the rational use of microbiota can serve as a new therapeutic strategy, diagnosis and prognosis evaluation for cancer and a potential therapeutic target for cancer therapy.

It is worth noting that intratumoral microbiota can regulate the immune microenvironment to mediate the outcome of tumors by promoting the inflammatory response or suppressing antitumor effects. Whether the intratumoral microbiota influences antitumor immunity depends on the microbiota composition, the crosstalk and interaction between the microbiota and cancer, and the status of cancers. Therefore, the depletion or enhancement of the microbiota in local tumor tissues should be carefully considered.

In recent years, the study of intratumoral microbiota has attracted more attention and made some progress, but current research is still limited. The mechanism of intratumoral microbiota affecting antitumor immunity and the efficacy of antitumor therapy is still unclear, which hinders the clinical application of microbial-related therapeutic strategies in tumors. Therefore, extensive validation through preclinical models and clinical trials is needed, and we believe that tumors can be successfully treated by administering microbiota, targeting microbiota, or combined with immunotherapy in the future.
